# Southern Medical College Commencement

**Published:** 1883-03-20

**Authors:** 


### EDITORIALS AND MISCELLANEOUS.

### *SOUTHERN MEDICAL COLLEGE COMMENCEMENT.*

The Commencement Exercises of the above new and rapidly rising Institution, took place at De Give's opera house on the evening of the 27th of February, 1883, and were of the most interesting character. The following account, given by one of our city papers, the *Monday Morning Mail*, is so well drawn that it will suffice to coyy it as published:

INTERESTING EXERCISES—THE SPEECHES,—NAMES OF GRADUATES—PRIZES, ETC. SOUTHERN MEDICAL COLLEGE.

On Tuesday night last, at De Give's, which was crowded, occurred the commencement exercises of this Institution, whose rapid growth to its present eminence is certainly a very high compliment to its originators and managers. After prayer by Rev. Geo. Leonard Chaney, began the evening's programme.

The annual report of Dr. W. P. Nicolson, Dean of the College, was as follows:

*Mr. President*—In making the annual report at the close of the session of 1883, it gives me much pleasure to say there is every cause for feelings of congratulation among the Trustees and Faculty, as the steps of progress have been, in many respects, very great. At the beginning of the term the obligation to the profession to which we stood committed, was fulfilled in the opening of the Central Ivy Street Hospital, adjoining the College, which was placed under the medical control of the Faculty by the Ladies' Hospital Association of this city. Many most interesting cases have been treated in this institution, which have been utilized as far as practicable for the benefit of the class. In the coming term we anticipate a much more extended supply of clinical material from this source, and pledge ourselves to use it faithfully and conscientiously for the imparting of medical instruction to our students.

Our schedule of lectures has been interrupted only by sickness, which, I regret to say, has, in one or two instances, laid its hand heavily upon us. It is with pain that I note the continued and serious illness of our Professor of the Principles and Practice of Surgery.

The matriculants of the school have numbered this session 108, and we present you for graduation this evening a class of thirty-seven. Py a singular coincidence the class numbers precisely the same as last session.

In conclusion, I would say that our graduating class to-night, by their high standing, have proved themselves worthy of any institution.

Prof. T. S. Powell, President of the Board of Trustees, then conferred the degree of Doctor of Medicine upon the following candidates for that honor: J. W. Albert, J. H. Alley, T. [illegible]. Appleby, W. A. Bradley, Gordon Carithers, D. R. Fluker, J. M. Fowler, W. L. Funderborg, C. M. George, J. P. Hall, D. F. Knott, W. A. McArthur, G. M. McMillan, J. B. Medlock, W. B. Clement, A. H. Crawford, A. I. Davis, A. S. Dyar, S. W. Everett, H. L. Harvey, J. T. Harwell, J. W. Howell, Lee Huffaker, G. W. Kelley, W. A. Monroe, J. A. Nelms, D.

R. Norman, E. P. Overby, F. B. Palmer, F. H. Sims, M. W. Speer, C. B. Thomas, M. R. Toland, F. Van Goldtsnoven, S. W. Visage, Jefferson Wilcox, and W. L. Wright.

Dr. Powell, having conferred the degrees, spoke as follows:

*Gentlemen of this, the Fourth Graduating Class of the Southern Medical College:*—As the President of this Institution, it is appropriate that I say a few parting words.

From this stage, as from a common haven, you begin the great voyage across the ocean of life. Your frail barks are gaily trimmed with fluttering hopes and ornamented with the glittering jewels of ever dreaming fancy. But, ah, my young professional brethren, how solemn are the reflections that arise while contemplating the scene and the occasion with the mind's eyes.

Where will we all be in the years to come? What will the course of each of you have been? Will it have been over a smooth sea, with favoring winds and happy prospects, or will the storms of adversity have beaten upon you?

Will danger be at the prow and sorrow at the helm? How far will each be on the voyage to the invisible shores of the spirit-land? How long before the breakers of death shall sound their dreadful warning? Will we all meet again in this pathless sea men call "LIFE," and hear from each other's lips the greeting, wafted across the dark billows,—"All's well?" Or shall we never meet until the beacon-light of eternity dawns upon the failing sight, and the haven of eternal rest opens its golden portals to receive the weary wanderer? Who can answer these questions, and yet you, we—all of us—ought to ask them in the profoundest humility.

Some of the answers are hidden by the impenetrable veil of the future, and the most important one can only be answered beyond the grave. Still this contemplation will teach us humility, one of the most precious ornaments of the mind, and without which all the knowledge gained from books, and all the arts and sciences taught by your skillful teachers shall avail you but little in the trials and experiences of life.

But, my dear Doctors, remember that whatever may befall you, the Heavenly Father above us, who teaches the hearts and souls of His children, will never forsake you, if you will listen to, and profit by the lessons of divine wisdom which He inculcates.

Remember, too, that in all the realm of memory there is no brighter spot for you than the one around which cluster the ever green recollections of your college days at the Southern Medical College.

Also remember there is nothing that conduces more to the happiness and peace of mind of the physician than the consciousness that he has done his duty well.

When the eve of life approaches and the setting sun beams its last ray over him, how pleasant it must be to feel that suffering humanity has been benefitted at his hands.

Remember, too, that there is something more ennobling—something of a greater value than the love of lucre, should actuate him to pursue the professional path he has chosen.

When the pallid sufferer lies groaning on his bed, the mere knowledge that by his suffering he would gain, should not induce him to attempt a cure.

Sympathy for his affliction should spring unbidden in his heart and the same feeling which would arise from the suffering of a brother should be engendered in him towards any fellow-creature, and he should endeavor to make his visits like stripes of bright, clear sky between the clouds. He should come like Aurora chasing away the darkness.

The howling storm, the chilling wintry winds, the scorching noontide sun, or the darkness of a moonless night, should not be an excuse for neglect.

In conclusion, my young brethren, let me entreat you to pursue the course you have been taught in this institution, and my word for it, success will be yours, and whether your life be robed in sunlight or darkness, by trouble and tempest, posterity will embalm your memory and a grateful world proclaim you a shining light in your own generation— a benefactor to successive generations of mankind.

The Rev. Henry McDonald then addressed the graduating class. He discussed the relations that ought to exist between the physician and the patient.

1. What the public has a right to expect of the physician: First. There must be the strong substratum of common sense. Second. There must be thorough training. Every State is recreant to its duty that does not require the young student to undergo thorough preparation. Third. There must be continual study. Follow up your studies, young men. There is a sort of a providence which gives a young Doctor plenty of time for keeping abreast of everything.

2. What has a physician the right to expect of the patient? First. Give the physician your confidence; call the physician on time. Many cases have been lost through delay in sending for the doctor, and in refusing him your confidence. Don't be misled. Beware of the Indian medicine man or the retired clergyman who peddles patent medicine. [Laughter.] It is surprising how much patent medicine a man can take —and live. [Great laughter.] Beware of clairvoyants or cure-alls. It is better to die in the orthodox ranks than to fall by the ruthless hand of the Goths and vandals. [Applause.] Second. Pay the Doctor; even if a man does work for the good of the cause, still a little money coming along occasionally helps a man to work with a better heart. Never, never fail to pay the Doctor. In conclusion, I wish you all well. May you all attain high rewards and succeed in getting good wives, live good lives, and at last reach that land where the inhabitants never say "I am sick." [Loud and continued applause.]

We have only touched some of the points in Dr. McDonald's address, which was very instructive and useful in its principles and grand and eloquent in sentiment and delivery.

Dr. Alexander S. Dyar, of Atlanta, delivered the valedictory. He spoke affectionately of the entire corps of professors, and of his cotemporary graduates, and acquitted himself with much credit. His reference to the great men of our times was well made, and his allusion to the lamented Ben. Hill, of Georgia, was eloquent and touching.

The presentation of prizes was made by Col. John H. Seals, of the *Sunny South*, as follows:

First Honor—Gold medal, value $50, offered by the Faculty for the highest aggregate examination, and was awarded to Dr. F. H. Sims, of Georgia.

Second Honor—Gold medal for next highest, by the Faculty, and was awarded to Dr. M. R. Toland, of Texas.

3. Gold medal offered by Prof. T. S. Powell, for highest examination in Obstetrics and Diseases of Women and Children, was awarded to Dr. F. H. Sims.

4. Gold medal, by Prof. G. G. Crawford, for highest examination upon Chemical, Operative and Scientific Surgery, was awarded to Dr. J. H Alley.

5. Gold medal, by Prof. W. D. Bizzell, for highest examination upon the Principles and Practice of Medicine, was awarded to Dr. C. B. Thomas.

6. Case containing Hypodermic Syringe and Clinical Thermometer, for highest examination in Materia Medica and Therapeutics, by Prof. G. G. Roy, was awarded to Dr. Jefferson Wilcox, of Georgia.

7. An Ophthalmascope, for highest examination upon Diseases of the Eye, Ear and Throat, by Prof. A. G. Hobbs, was awarded to Dr. C. B. Thomas.

8. Medical Case, by Prof. R. C. Word, for highest examination in Physiology. Awarded to Dr. M. R. Toland.

9. A case of instruments, by Prof. Wm. Perrin Nicolson, for highest examination upon General and Descriptive Anatomy, awarded to Dr. M. R. Toland.

10. Chemical Set, by Prof. Burns, for highest examination in Chemistry. Awarded to Dr. F. H. Sims.

The benediction was pronounced by the Rev. Mr. Chaney.

Wurm's orchestra discoursed sweet music at intervals.

The ladies manifested great interest in the exercises, casting boquets to the graduates and warmly participating in the plaudits of the occasion.

